# Impact of non-emergency surgical timing on postoperative recovery quality in mild or asymptomatic SARS-CoV-2 infected patients: a grouped cohort study

**DOI:** 10.1186/s12871-024-02600-y

**Published:** 2024-07-06

**Authors:** Qiu-Bo Wang, Yu-Long Wang, Yue-Feng Wang, Hua Chen, Wei Chen, Yong-Quan Chen

**Affiliations:** 1 Department of Anaesthesiology, Yijishan Hospital, Wannan Medical College, Wuhu, 241000 China; 2 Department of Anaesthesiology, Bozhou Traditional Chinese Medicine Hospital, Bozhou, 236800 China

**Keywords:** SARA-CoV-2, Omicron, Postoperative recovery quality, Surgical timing, General anaesthesia, Quality of recovery-15 scale

## Abstract

**Objective:**

To explore the relationship between the timing of non-emergency surgery in mild or asymptomatic SARS-CoV-2 (severe acute respiratory syndrome coronavirus 2) infected individuals and the quality of postoperative recovery from the time of confirmed infection to the day of surgery.

**Methods:**

We retrospectively reviewed the medical records of 300 cases of mild or asymptomatic SARS-CoV-2 infected patients undergoing elective general anaesthesia surgery at Yijishan Hospital between January 9, 2023, and February 17, 2023. Based on the time from confirmed SARS-CoV-2 infection to the day of surgery, patients were divided into four groups: ≤2 weeks (Group A), 2–4 weeks (Group B), 4–6 weeks (Group C), and 6–8 weeks (Group D). The primary outcome measures included the Quality of Recovery-15 (QoR-15) scale scores at 3 days, 3 months, and 6 months postoperatively. Secondary outcome measures included postoperative mortality, ICU admission, pulmonary complications, postoperative length of hospital stay, extubation time, and time to leave the PACU.

**Results:**

Concerning the primary outcome measures, the QoR-15 scores at 3 days postoperatively in Group A were significantly lower compared to the other three groups (*P* < 0.05), while there were no statistically significant differences among the other three groups (*P* > 0.05). The QoR-15 scores at 3 and 6 months postoperatively showed no statistically significant differences among the four groups (*P* > 0.05). In terms of secondary outcome measures, Group A had a significantly prolonged hospital stay compared to the other three groups (*P* < 0.05), while other outcome measures showed no statistically significant differences (*P* > 0.05).

**Conclusion:**

The timing of surgery in mild or asymptomatic SARS-CoV-2 infected patients does not affect long-term recovery quality but does impact short-term recovery quality, especially for elective general anaesthesia surgeries within 2 weeks of confirmed infection. Therefore, it is recommended to wait for a surgical timing of at least greater than 2 weeks to improve short-term recovery quality and enhance patient prognosis.

## Introduction

Surgery stands as the primary and most efficacious therapeutic intervention for disease management. Perioperative safety is a universally shared concern. Medical teams, particularly anaesthetists, shoulder significant responsibilities in anticipating and mitigating perioperative risks, necessitating proficiency in assessment and management. However, during unforeseen public health emergencies, numerous risk factors necessitate validation through investigative studies to provide evidence for clinical practice.

Since the first confirmed case of Severe Acute Respiratory Syndrome Coronavirus 2 (SARS-CoV-2) infection in 2019, as of December 1, 2022, the global reach of the Corona Virus Disease 2019 (COVID-19) pandemic has impacted over 200 countries and regions, with over 643 million confirmed cases and more than 6.63 million deaths. Individuals infected with SARS-CoV-2 may exhibit short-term and long-term multi-systemic sequelae [1–5], resulting in multi-organ damage and, in severe instances, mortality [6–12]. In November 2021, the SARS-CoV-2 Omicron variant was identified in South Africa [13], rapidly disseminating, and by December 2022, it emerged as the predominant strain causing infections in Anhui Province, China. Among the numerous variants during the COVID-19 pandemic, Omicron displays the highest mutation count, marked by high transmissibility and lower pathogenicity, leading to significant reductions in hospitalization, severity, and mortality rates [14–16]. In response to the evolving situation, China classified SARS-CoV-2 infection as a national category B infectious disease on January 8, 2023. With the easing of stringent epidemic control measures, there has been a notable increase in individuals infected with the Omicron variant awaiting surgery.

SARS-CoV-2 infection significantly heightens perioperative risks for patients. While early expert consensus suggested that most mild or asymptomatic SARS-CoV-2-infected individuals could undergo surgery after 7 weeks of diagnosis (except in cases of clinical urgency and risks surpassing the progression of the disease) [17], the substantial number of patients requiring timely surgery not only imposes a burden on patients during the 7-week waiting period but also significantly increases the healthcare load on society. Furthermore, given the distinctive pathogenic characteristics of Omicron differing markedly from previous strains, investigating the appropriate timing for surgery holds paramount significance.

This study investigates the short-term and long-term postoperative recovery quality of SARS-CoV-2-infected individuals at Yijishan Hospital, Wannan Medical College, considering the widespread transmission of the Omicron variant and post-COVID-19 vaccination. The aim is to examine the impact of different timings for surgery under general anaesthesia on the postoperative recovery quality in SARS-CoV-2-infected individuals.

## Materials and methods

Following ethical approval by the Ethics Committee of Yijishan Hospital of Wannan Medical College (Protocol Number: 2023-68) and with waiver of individual written informed consent, we conducted a retrospective cohort study including patients who had elective general anaesthesia surgery at Yijishan Hospital from January 9, 2023, to February 17, 2023. The inclusion criteria were: patients aged 18 to 75 years, with an American Society of Anaesthesiologists (ASA) classification of I to III, who had been confirmed as having a mild or asymptomatic SARS-CoV-2 infection at least 8 weeks prior to surgery. Confirmation of infection was based on the positive detection of viral nucleic acid from nasal or throat swab antigen tests or reverse transcription-polymerase chain reaction (RT-PCR) for the novel coronavirus. Exclusion criteria included: individuals with mental disorders, those with severe dysfunction of the heart, lungs, liver, or kidneys, and individuals with severe hearing or communication impairments.

Data were extracted by reviewing the electronic medical records from the hospital’s information system, which included age, gender, body mass index (BMI), hypertension, diabetes, chest computed tomography (CT) findings with inflammation, ASA classification, grade of surgery, operation time, potential of hydrogen (PH), oxygenation index, intraoperative bleeding, intraoperative fluid replacement, incidence of intraoperative use of vasoactive drugs, intensive care unit (ICU) admission, extubation time, time to leave the post-anaesthesia care unit (PACU), postoperative length of hospital stay (day 0 defined as the day of surgery), postoperative complications, and the QoR-15 scale scores at 3 days, 3 months, and 6 months postoperatively.

### Anaesthetic procedure

All patients underwent routine fasting and abstinence from liquids before surgery. Upon admission, all patients received standard anaesthetic management, with routine monitoring of non-invasive blood pressure (BP), heart rate (HR), pulse oxygen saturation (SpO_2_), temperature (T), and the establishment of upper limb venous access for intravenous infusion of electrolyte solution at 6–8 ml/(kg·h). Anaesthetic induction involved the intravenous administration of midazolam 0.02–0.03 mg/kg, sufentanil 0.3–0.4 µg/kg, etomidate 0.2–0.3 mg/kg, and cisatracurium 0.15–0.2 mg/kg. After meeting intubation criteria, patients received endotracheal intubation or a laryngeal mask, with ventilation set to controlled volume ventilation. Respiratory parameters were adjusted with oxygen flow at 2 L/min, tidal volume (V_T_) at 6–8 ml/kg, respiratory rate at 12–14 breaths/min, maintaining PETCO2 at 35–45 mmHg, and FiO2 at 100%. Anaesthetic maintenance involved intravenous anaesthesia, with propofol at 4–8 mg/(kg·h), remifentanil at 0.1–0.2 µg/(kg·min), and intermittent injections of cisatracurium at 2–4 mg. Bispectral index (BIS) depth monitoring was maintained between 40 and 60, and arterial blood gas analysis was conducted 15 min after anaesthetic maintenance initiation. Throughout the anaesthetic process, mean arterial pressure (MAP) was maintained within ± 20% of baseline, HR between 60 and 100 beats/min, and body temperature between 36 and 37 °C. After surgery, anaesthetic agents were discontinued, and patients were transferred to the PACU or ICU. Tracheal tubes or laryngeal masks were removed upon meeting extubation criteria, and patients meeting transfer standards were returned to the ward.

### Main outcome measures

The primary outcome measures comprised QoR-15 scale scores on postoperative day 3, postoperative month 3, and postoperative month 6. Secondary outcome measures included endotracheal tube removal time, PACU departure time, length of hospital stay (day 0 defined as the day of surgery), ICU admission, postoperative mortality, pulmonary infections, respiratory failure, sepsis, and fatigue incidence.

### Statistical methods

Statistical analyses were performed using SPSS 18.0 (SPSS Inc., Chicago, IL, USA). Normally distributed continuous data were expressed as mean ± standard deviation, with intergroup comparisons conducted through one-way analysis of variance. Non-normally distributed continuous data were presented as median (25th percentile-75th percentile), and intergroup comparisons were executed using the Kruskal-Wallis test. Adjusted pairwise comparisons for *P*-values were performed with Bonferroni correction, followed by the Mann-Whitney U test. Count data were reported as frequency/percentage (%), and intergroup comparisons were analysed using the chi-squared test. The significance level was set at α = 0.05, with *P* < 0.05 indicating statistical significance.

## Results

### Participants

**Fig. 1:**
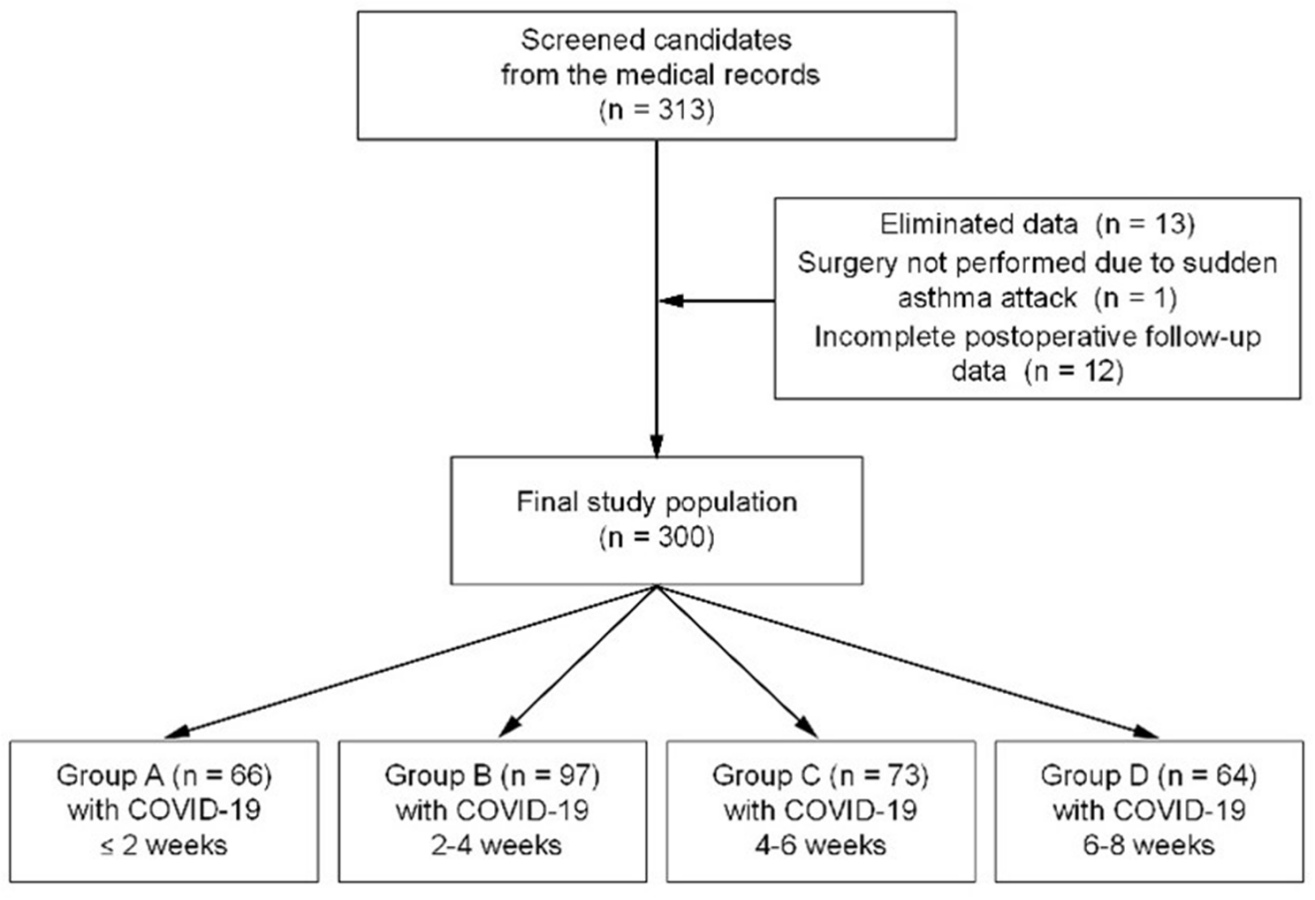
Technology roadmap

Based on the inclusion and exclusion criteria, a preliminary screening of electronic medical records yielded 313 eligible patients. Further review of anesthesia records and postoperative follow-up revealed that one patient’s surgery was aborted due to an unexpected asthma attack, and that 12 patients had incomplete postoperative follow-up data. Consequently, data from these 13 patients were eliminated, ultimately leaving a total of 300 patients in the study. Then patients’ data have been categorized by the time interval between the diagnosis of initial SARS-CoV-2 infection and the surgery, as depicted in Fig. 1.

### Demographic data

There were no statistically significant differences (*P* > 0.05) among the four groups concerning patients’ age, gender, BMI, hypertension, diabetes, preoperative chest CT, ASA classification, surgical complexity, operative duration, arterial blood PH, oxygenation index, intraoperative bleeding, intraoperative fluid replacement and incidence of intraoperative use of vasoactive drugs. The specifics are delineated in Table 1.

**Table 1:** Comparative analysis of patients’ general characteristics

Variable	Group A (*n* = 66)	Group B (*n* = 97)	Group C (*n* = 73)	Group D (*n* = 64)	*P* value
Age (y)	53 (44–61)	52 (41–59)	51 (39–60)	53 (42–60)	0.590
Gender (male/female)	33/33	33/64	31/42	28/36	0.229
BMI (kg/m^2^)	23.5 (21.2–26.5)	23.4 (21.0-26.2)	23.9 (21.3–25.6)	24.7 (22.2–26.2)	0.584
Hypertension (%)	20 (30.3)	19 (19.6)	17 (23.3)	21 (32.8)	0.208
Diabetes (%)	7 (10.6)	4 (4.1)	8 (11.0)	5 (7.8)	0.326
Chest CT with inflammation (%)	11 (16.7)	15 (15.5)	13 (17.8)	11 (17.2)	0.980
ASA I-II/III	57/9	84/13	67/6	57/7	0.704
Grade of surgery 1–2/3–4	6/60	12/85	10/63	4/60	0.479
Operation time (min)	91 (64–144)	100 (55–139)	90 (55–125)	86 (53–134)	0.697
PH	7.308 ± 0.035	7.316 ± 0.049	7.371 ± 0.043	7.381 ± 0.042	0.330
Oxygenation index(mmHg)	374 ± 93	385 ± 94	387 ± 83	382 ± 74	0.819
Intraoperative bleeding (ml)	30 (9–70)	30 (5–80)	40 (18–100)	30 (15–60)	0.468
Intraoperative fluid replacement (ml)	1000 (1000–1500)	1000 (1000–1500)	1000 (1000–1500)	1000 (1000–1075)	0.099
Incidence of intraoperative use of vasoactive drugs (%)	3 (4.5)	5 (5.2)	2 (2.7)	2 (3.1)	0.879

Age, BMI, Operation time, Intraoperative bleeding and Intraoperative fluid replacement were presented as median (interquartile ranges: 25th percentile-75th percentile); PH and Oxygenation index were presented as the mean ± standard deviation; other continuous variables were represented as the number (percent). *P* value tested by the independent-sample t test, rank sum test or chi-square test, according to the specific situation

### QoR-15 scale scores on postoperative day 3, month 3 and month 6

**Fig. 2:**
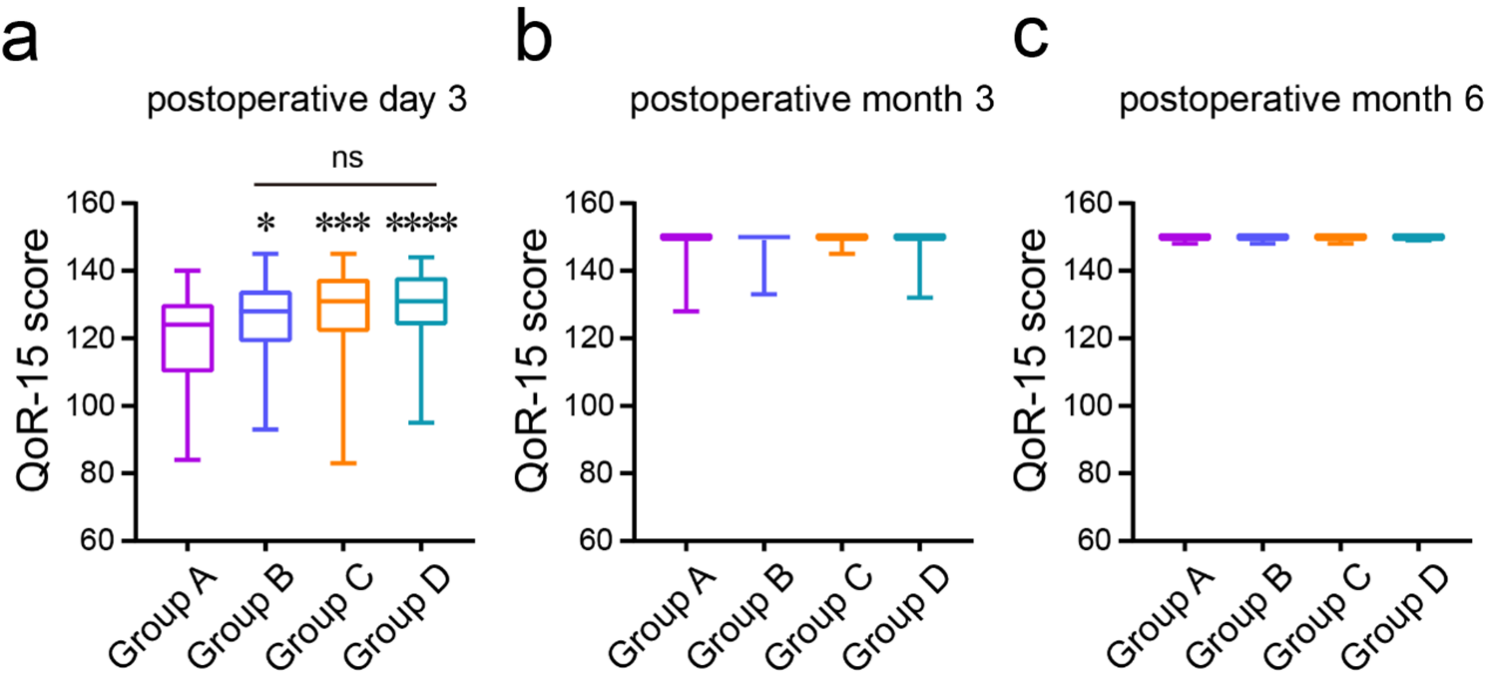
Comparative analysis of QoR-15 scale scores among four patient groups. QoR-15 is a 15-item scale for evaluating recovery quality. In comparison to Group A, *P < 0.05, ***P and ****P < 0.001. Pairwise comparisons between Groups B, C, and D, with ^ns^*P* > 0.05

On postoperative day 3, Group A exhibited consistently lower median QoR-15 scores [124 (110~129)] compared to Group B [128 (119~134)], Group C [131 (122~138)], and Group D [131 (124~138)] (*P* < 0.05) (Fig. 2a). However, at postoperative months 3 and 6, no statistically significant differences in QoR-15 scores were observed among the four groups (*P* > 0.05) (Fig. 2b, c).

To identify whether the surgical complexity and the ASA grade have any influence on the QoR-15 scores of postoperative day 3, we stratified comparison. No statistically significant differences (*P* > 0.05) were noted in the subgroup Grade of surgery 1–2 compared to the subgroup Grade of surgery 3–4, as outlined in Table 2. However, the patients accessed as ASA III have a lower QoR-15 scores in the group A (*P* = 0.003) and group B (*P* = 0.007), compared with the ASA I-II group, while there is no significant difference both in the group C and D (*P* > 0.05), as outlined in Table 3.

**Table 2:** The QoR-15 scale scores of the subgroup based on surgery grade on postoperative day 3

Variable	Grade of surgery 1–2	Grade of surgery 3–4	*P* value
Group A	138 (99–139)	124 (110–129)	0.118
Group B	134 (129–137)	127 (118–134)	0.165
Group C	135 (130–137)	130 (121–138)	0.183
Group D	137 (122–140)	131 (124–138)	0.367

Values presented as median (interquartile ranges: 25th percentile-75th percentile). *P* value tested by the independent-sample *t* test

**Table 3:** The QoR-15 scale scores of the ASA I-II and ASA III subgroups on postoperative day 3

Variable	ASA I-II	ASA III	*P* value
Group A	124 (119–131)	108 (99–118) *	0.003
Group B	129 (121–135)	113 (107–129) *	0.007
Group C	131 (122–136)	135 (125–141)	0.296
Group D	131 (124–138)	132 (126–138)	0.682

Values presented as median (interquartile ranges: 25th percentile-75th percentile). *P* value tested by the independent-sample *t* test, *P < 0.05

### Patient outcomes

Hospital Stay, Adverse Postoperative Outcomes, Extubation Time, and PACU Departure Time: In contrast to Group A, patients in Group B, C, and D demonstrated significantly shorter median hospital stays (*P* < 0.05). No statistically significant differences (*P* > 0.05) were noted among the four groups concerning Postoperative Mortality, ICU Admission, Pulmonary Infections, Respiratory Failure, Sepsis, Fatigue Incidence, Extubation time, and PACU departure Time, as outlined in Table 4.

**Table 4:** Comparative analysis of secondary outcome measures among four group

Variable	Group A (*n* = 66)	Group B (*n* = 97)	Group C (*n* = 73)	Group D (*n* = 64)	*P* value
ICU Admission (%)	2 (3)	2 (2.1)	1 (1.4)	0 (0)	0.410
Extubation time (min)	16 (10–25)	16 (10–27)	15 (10–23)	18 (12–25)	0.399
PACU departure time (min)	36 (23–48)	32 (25–43)	32 (25–44)	32 (27–38)	0.516
Hospital Stay (d)	6 (4–8)	4 (3–6) *	4 (3–6) *	4 (3–7) *	0.006
Postoperative Complications					
Death (%)	1 (1.5)	0 (0)	1 (1.4)	0 (0)	0.377
Pulmonary infections (%)	1 (1.5)	0 (0)	0 (0)	0 (0)	0.385
Respiratory failure (%)	1 (1.5)	0 (0)	0 (0)	0 (0)	0.385
Fatigue (%)	4 (6.1)	3 (3.1)	2 (2.7)	2 (3.1)	0.742
Sepsis (%)	1 (1.5)	0 (0)	0 (0)	0 (0)	0.385

Extubation time, PACU departure time and Hospital Stay were presented as median (interquartile ranges: 25th percentile-75th percentile); other continuous variables were represented as the number (percent). *P* value tested by rank sum test or chi-square test, according to the specific situation. *P < 0.05 compared to Group A.

## Discussion

Against the backdrop of widespread COVID-19 vaccination and the emergence of the Omicron variant, our study delved into the correlation between short- and long-term recovery quality and the timing of surgery in patients undergoing general anaesthesia post-SARS-CoV-2 infection. We analysed the data from a six-month follow-up with patients diagnosed with mild or asymptomatic SARS-CoV-2 infection. Notably, the group with an infection duration of less than two weeks displayed diminished QoR-15 scores three days postoperatively and an extended hospital stay. Our findings suggest that scheduling elective general anaesthesia surgery more than two weeks after the diagnosis of mild or asymptomatic SARS-CoV-2 infection may be more favourable for enhancing postoperative recovery quality.

The QoR-15 scale, introduced in 2013 for assessing overall anaesthesia and postoperative recovery quality [18], provides a comprehensive and more efficient evaluation compared to the QoR-40 scale. It is presently endorsed as a standard measurement for clinical trial outcomes [19]. With a total score of 150, higher scores signify superior postoperative recovery quality, and scores of 118 or above are indicative of a good recovery [20]. Our study indicates that the majority of patients, 80% on day 3, and 99% at 3 and 6 months postoperatively, achieved QoR-15 scores of 118 or above, indicating good postoperative recovery quality. However, the group with an infection duration of less than two weeks exhibited significantly lower QoR-15 scores at three days postoperatively, consistent with prior research [21, 22]. This may be attributed to inconsistent waiting times post-SARS-CoV-2 infection and anaesthetic agents known to suppress the body’s immune function, potentially prolonging active viral replication in patients with compromised immunity, thus slowing recovery from SARA-CoV-2 [23]. Research indicates that patients infected with the Omicron variant within eight weeks before surgery experience a shorter postoperative hospital stay and a significantly reduced risk of adverse postoperative outcomes [24–26]. This aligns with our findings, where the group with an infection duration of less than two weeks exhibited a significantly longer median hospital stay, aligning with the results of QoR-15 scores three days postoperatively. The consistency between these findings underscores the reliability of study results, emphasizing that undergoing surgery shortly after SARS-CoV-2 infection is not conducive to enhancing recovery quality. SARS-CoV-2 infection may be associated with short-term and long-term multi-system sequelae, including chronic pulmonary dysfunction, cognitive impairment, psychological distress, chronic fatigue, etc. [1–5]. Our study identified one patient in Group A who died due to respiratory failure combined with hepatic and renal failure, one readmitted for sepsis, and four reporting fatigue with slight exertion. In Group B, three patients reported slight fatigue with movement, while in Group C, one patient died due to cancer spread, two reported fatigue with slight exertion. In Group D, two patients reported slight fatigue with movement, consistent with previous reports. However, comparisons among the groups revealed no statistically significant differences. Our study has not observed a significant impact of Omicron variant infection on postoperative survival rates, contrary to previous research indicating an increased risk of postoperative mortality following SARS-CoV-2 infection. Possible reasons include: first, our study focused on patients with mild or asymptomatic infection; second, the low pathogenicity of the Omicron variant itself [27–31]; third, widespread vaccination in China; fourth, previous studies were conducted before the Omicron variant became the dominant strain [32]. Investigating the postoperative short- and long-term sequelae of SARS-CoV-2 infection in Omicron variant-infected individuals with expanded inclusion remains clinically significant. Previous studies primarily explored postoperative mortality and pulmonary complication rates in SARS-CoV-2-infected patients, often conducted before the emergence of the Omicron variant and widespread vaccination, making some findings less applicable to the current scenario. The Omicron variant primarily affects the upper respiratory tract, with most infected individuals exhibiting normal chest CT scans [27–31, 33], resulting in a significant reduction in mortality and pulmonary complications 30 days postoperatively. Therefore, exploring postoperative recovery quality holds greater clinical significance.

We stratified comparison and found that the ASA grade is more sensitive than surgical grade on influencing the quality of recovery. The possible reasons may be as follows: Firstly, the sample size is too small to present significant difference between in the subgroups of surgery grade 1–2 and 3–4 on the QoR-15 scores; Secondly, the surgical grading is based on technical difficulty, complexity and risk of surgery, and a high surgical grade does not mean a high surgical trauma to patients, especially the rapid development of minimally invasive surgery in recent years; Finally, the ASA grading may be more comprehensive, considering not only the surgery grade but also general condition, including the SARS-CoV-2-infected, of the patient observed before anaesthesia. However, we cannot conclude whether the timing of surgery based on the duration of SARS-CoV-2 infection or the grade of surgery has a greater impact on the quality of postoperative recovery in the study. Because, in the precondition with no difference at the proportions of surgery grades between the subgroups, we only explored the influence of the timing of surgery after SARS-CoV-2 infection on the quality of postoperative recovery.

This study has several limitations. Firstly, being a single-center study, the sample size is limited, and the surveyed population is regionally constrained. Larger-scale studies are required for validation. Secondly, some patients with a history of SARS-CoV-2 infection might have been misclassified as never infected. This is particularly plausible for asymptomatic patients who might not have undergone nucleic acid testing and, consequently, were not included in this study. Additionally, our study did not include an uninfected group, lacking a blank control. Lastly, this study is based on the time from SARS-CoV-2 diagnosis to the day of surgery, but delays in diagnosis might have occurred in some patients, potentially underestimating the true interval between infection and surgery.

In summary, this article examines the prognosis of elective general anaesthesia surgery at different timings in patients with mild or asymptomatic SARS-CoV-2 infection. Despite the relatively low pathogenicity of the Omicron variant, immunosuppression is prevalent in surgical patients. The group with an infection duration of fewer than two weeks undergoing non-emergency general anaesthesia surgery showed a decreased short-term postoperative recovery quality, prolonged hospital stay, and increased risk of postoperative complications, without affecting long-term postoperative recovery quality and postoperative mortality. Therefore, we recommend that patients with mild or asymptomatic SARS-CoV-2 infection wait for more than two weeks before undergoing non-emergency general anaesthesia surgery, as it proves to be a more advantageous choice.

## Data Availability

The datasets generated and/or analysed during this study are not publicly available due to institutional policy on data confidentiality but are available from the corresponding author on reasonable request.

## Abbreviations

ASA: American Society of Anaesthesiologists
BIS: Bispectral index
BMI: Body Mass Index
BP: Blood Pressure
CT: Computed Tomography
COVID-19: Corona Virus Disease 2019
ECG: Electrocardiogram
FiO_2_: Fraction of Inspiration O_2_
HR: Heart Rate
ICU: Intensive Care Unit
MAP: Mean Arterial Pressure
PACU: Post-Anaesthesia Care Unit
PH: Potential of Hydrogen
P_ET_CO_2_: Peak Expiratory CO_2_ Pressure
SpO2: Pulse Oxygen Saturation
T: Temperature
SARS-CoV-2: Severe Acute Respiratory Syndrome Coronavirus 2
V_T_: Tidal Volume

## References

[1] Huang C, Huang L, Wang Y, et al. 6-month consequences of COVID-19 in patients discharged from hospital: a cohort study[J]. Lancet, 2021, 397(10270): 220–232. 10.1016/S0140-6736(20)32656-8 PMC7833295 33428867

[2] Zhao Y M, Shang Y M, Song W B, et al. Follow-up study of the pulmonary function and related physiological characteristics of COVID-19 survivors three months after recovery[J]. EClinicalMedicine, 2020, 25: 100463. 10.1016/j.eclinm.2020.100463 PMC7361108 32838236

[3] Carfì A, Bernabei R, Landi F. Persistent Symptoms in Patients After Acute COVID-19[J]. Jama, 2020, 324(6): 603–605. 10.1001/jama.2020.12603 PMC7349096 32644129

[4] Ceban F, Ling S, Lui L M W, et al. Fatigue and cognitive impairment in Post-COVID-19 Syndrome: A systematic review and meta-analysis[J]. Brain Behav Immun, 2022, 101: 93–135. 10.1016/j.bbi.2021.12.020 PMC8715665 34973396

[5] Mahmoud Z, East L, Gleva M, et al. Cardiovascular symptom phenotypes of post-acute sequelae of SARS-CoV-2[J]. Int J Cardiol, 2022, 366: 35–41. 10.1016/j.ijcard.2022.07.018 PMC9278009 35842003

[6] Han X, Ye Q. Kidney involvement in COVID-19 and its treatments[J]. J Med Virol, 2021, 93(3): 1387–1395. 10.1002/jmv.26653 33150973

[7] Tian D, Ye Q. Hepatic complications of COVID-19 and its treatment[J]. J Med Virol, 2020, 92(10): 1818–1824. 10.1002/jmv.26036 PMC7280725 32437004

[8] Ye Q, Lu D, Shang S, et al. Crosstalk between coronavirus disease 2019 and cardiovascular disease and its treatment[J]. ESC Heart Fail, 2020, 7(6): 3464–3472. 10.1002/ehf2.12960 PMC7754975 32935928

[9] Luo H C, You C Y, Lu S W, et al. Characteristics of coagulation alteration in patients with COVID-19[J]. Ann Hematol, 2021, 100(1): 45–52. 10.1007/s00277-020-04305-x PMC7572245 33079220

[10] Ye Q, Wang B, Zhang T, et al. The mechanism and treatment of gastrointestinal symptoms in patients with COVID-19[J]. Am J Physiol Gastrointest Liver Physiol, 2020, 319(2): G245-g252. 10.1152/ajpgi.00148.2020 PMC7414235 32639848

[11] Gupta A, Madhavan M V, Sehgal K, et al. Extrapulmonary manifestations of COVID-19[J]. Nat Med, 2020, 26(7): 1017–1032. 10.1038/s41591-020-0968-3 PMC11972613 32651579

[12] Chen N, Zhou M, Dong X, et al. Epidemiological and clinical characteristics of 99 cases of 2019 novel coronavirus pneumonia in Wuhan, China: a descriptive study[J]. Lancet, 2020, 395(10223): 507–513. 10.1016/S0140-6736(20)30211-7 PMC7135076 32007143

[13] Classification of Omicron (B.1.1.529): SARS-CoV-2 Variant of Concern[EB/OL]. [2023-06-01]. https://www.who.int/news/item/26-11-2021-classification-of-omicron-(b.1.1.529)-sars-cov-2-variant-of-concern.

[14] Xu R, Wang W, Zhang W. As the SARS-CoV-2 virus evolves, should Omicron subvariant BA.2 be subjected to quarantine, or should we learn to live with it?[J]. Front Public Health, 2022, 10: 1039123. 10.3389/fpubh.2022.1039123 PMC9730036 36504951

[15] Fan Y, Li X, Zhang L, et al. SARS-CoV-2 Omicron variant: recent progress and future perspectives[J]. Signal Transduct Target Ther, 2022, 7(1): 141. 10.1038/s41392-022-00997-x PMC9047469 35484110

[16] Halfmann P J, Iida S, Iwatsuki-Horimoto K, et al. SARS-CoV-2 Omicron virus causes attenuated disease in mice and hamsters[J]. Nature, 2022, 603(7902): 687–692. 10.1038/s41586-022-04441-6 PMC8942849 35062015

[17] El-Boghdadly K, Cook T M, Goodacre T, et al. SARS-CoV-2 infection, COVID-19 and timing of elective surgery: A multidisciplinary consensus statement on behalf of the Association of Anaesthetists, the Centre for Peri-operative Care, the Federation of Surgical Specialty Associations, the Royal College of Anaesthetists and the Royal College of Surgeons of England[J]. Anaesthesia, 2021, 76(7): 940–946. 10.1111/anae.15464 PMC8250763 33735942

[18] Stark P A, Myles P S, Burke J A. Development and psychometric evaluation of a postoperative quality of recovery score: the QoR-15[J]. Anaesthesiology, 2013, 118(6): 1332-40. 10.1097/ALN.0b013e318289b84b 23411725

[19] Kleif J, Waage J, Christensen K B, et al. Systematic review of the QoR-15 score, a patient- reported outcome measure measuring quality of recovery after surgery and anaesthesia[J]. Br J Anaesth, 2018, 120(1): 28–36. 10.1016/j.bja.2017.11.013 29397134

[20] Myles P S, Shulman M A, Reilly J, et al. Measurement of quality of recovery after surgery using the 15-item quality of recovery scale: a systematic review and meta-analysis[J]. Br J Anaesth, 2022, 128(6): 1029–1039. 10.1016/j.bja.2022.03.009 35430086

[21] Mortality and pulmonary complications in patients undergoing surgery with perioperative SARS-CoV-2 infection: an international cohort study[J]. Lancet, 2020, 396(10243): 27–38. 10.1016/S0140-6736(20)31182-X PMC7259900 32479829

[22] Outcomes and Their State-level Variation in Patients Undergoing Surgery With Perioperative SARS-CoV-2 Infection in the USA: A Prospective Multicenter Study[J]. Ann Surg, 2022, 275(2): 247–251. 10.1097/SLA.0000000000005310 PMC8745946 34793350

[23] Ackerman R S, Luddy K A, Icard B E, et al. The Effects of Anaesthetics and Perioperative Medications on Immune Function: A Narrative Review[J]. Anaesth Analg, 2021, 133(3): 676–689. 10.1213/ANE.0000000000005607 34100781

[24] Le S T, Kipnis P, Cohn B, et al. COVID-19 Vaccination and the Timing of Surgery Following COVID-19 Infection[J]. Ann Surg, 2022, 276(5): e265-e272. 10.1097/SLA.0000000000005597 PMC9533764 35837898

[25] Prasad N K, Lake R, Englum B R, et al. COVID-19 Vaccination Associated With Reduced Postoperative SARS-CoV-2 Infection and Morbidity[J]. Ann Surg, 2022, 275(1): 31–36. 10.1097/SLA.0000000000005176 PMC8678152 34417362

[26] Garnier M, Constantin J M, Cinotti R, et al. Association of preoperative COVID-19 and postoperative respiratory morbidity during the Omicron epidemic wave: the DROMIS-22 multicentre prospective observational cohort study[J]. EClinicalMedicine, 2023, 58: 101881. 10.1016/j.eclinm.2023.101881 PMC9975647 36873425

[27] Menni C, Valdes A M, Polidori L, et al. Symptom prevalence, duration, and risk of hospital admission in individuals infected with SARS-CoV-2 during periods of omicron and delta variant dominance: a prospective observational study from the ZOE COVID Study[J]. Lancet, 2022, 399(10335): 1618–1624. 10.1016/S0140-6736(22)00327-0 PMC8989396 35397851

[28] Jassat W, Abdool Karim S S, Mudara C, et al. Clinical severity of COVID-19 in patients admitted to hospital during the omicron wave in South Africa: a retrospective observational study[J]. Lancet Glob Health, 2022, 10(7): e961-e969. 10.1016/S2214-109X(22)00114-0 PMC9116895 35597249

[29] Davies M A, Kassanjee R, Rousseau P, et al. Outcomes of laboratory-confirmed SARS-CoV-2 infection in the Omicron-driven fourth wave compared with previous waves in the Western Cape Province, South Africa[J]. Trop Med Int Health, 2022, 27(6): 564–573. 10.1111/tmi.13752 PMC9115442 35411997

[30] Ulloa A C, Buchan S A, Daneman N, et al. Estimates of SARS-CoV-2 Omicron Variant Severity in Ontario, Canada[J]. Jama, 2022, 327(13): 1286–1288. 10.1001/jama.2022.2274 PMC8855311 35175280

[31] Abdullah F, Myers J, Basu D, et al. Decreased severity of disease during the first global omicron variant covid-19 outbreak in a large hospital in tshwane, south africa[J]. Int J Infect Dis, 2022, 116: 38–42. 10.1016/j.ijid.2021.12.357 PMC8713416 34971823

[32] Timing of surgery following SARS-CoV-2 infection: an international prospective cohort study[J]. Anaesthesia, 2021, 76(6): 748–758. 10.1111/anae.15458 PMC8206995 33690889

[33] Tsakok M T, Watson R A, Saujani S J, et al. Reduction in Chest CT Severity and Improved Hospital Outcomes in SARS-CoV-2 Omicron Compared with Delta Variant Infection[J]. Radiology, 2023, 306(1): 261–269. 10.1148/radiol.220533 PMC9272784 35727150

